# Prevalence of Colorectal Polyps Based on Cardiorespiratory Fitness, Muscle Strength, Health Behavior, and Abdominal Obesity in Asymptomatic Elderly

**DOI:** 10.3390/healthcare9101400

**Published:** 2021-10-19

**Authors:** Shiyu Zhang, Junyong Zhang, Yonghwan Kim, Wangyang Zhang

**Affiliations:** 1 School of Physical Education in Main Campus, Zhengzhou University, Zhengzhou 450001, China; zsy0628@gs.zzu.edu.cn (S.Z.); zzdxfcy@gs.zzu.edu.cn (J.Z.); 2 Department of Physical Education, Gangneung-Wonju National University, Gangneung 25457, Korea; yhkim@gwnu.ac.kr; 3 School of Physical Education in Main Campus, Postdoctoral Mobile Station of Public Administration, Zhengzhou University, Zhengzhou 450001, China

**Keywords:** colorectal polyp, cardiorespiratory fitness, muscle strength, health behavior, prevalence

## Abstract

Colorectal polyps are precursor lesions of colorectal cancer and are known to be associated with obesity, low physical activity, and unhealthy behavior. This cross-sectional study analyzed the prevalence of colorectal polyps based on cardiorespiratory fitness (CRF), muscle strength, and health behavior in older adults. Participants were asymptomatic and included 1024 men and 472 women aged 65–80 years who visited the health care center. Colonoscopy was performed under conscious sedation, and cardiorespiratory fitness was measured as the maximum oxygen uptake using gas analysis. Muscle strength was determined using isokinetic equipment, and physical activity, alcohol consumption, and smoking status were investigated using questionnaires. Waist circumference was measured at the thickest part of the middle of the abdomen. Logistic regression analysis was used to calculate the prevalence of colorectal polyps using odds ratios (ORs) based on the variables. The incidence of colorectal polyps was 65.2% in men and 48.5% in women. The ORs of colorectal polyps for obesity were 1.151 (95% confidence interval [CI], 1.010–2.291) and 1.178 (95% CI, 1.015–2.612) in men and women, respectively. The OR for colorectal polyps in male current smokers was 1.884. The ORs for low CRF were 1.985 and 1.841 in men and women, respectively, compared with high CRF. The prevalence of polyps increased with low muscle strength (men’s OR 1.343 women’s OR 1.440) and physical activity in both men (OR 1.693) and women (OR 1.861). In conclusion, lower CRF and muscle strength were associated with an increased prevalence of colorectal polyps in men and women. In both sexes, high waist circumference and low physical activity increased the prevalence of colorectal polyps.

## 1. Introduction

Cancer is a leading cause of mortality worldwide, and according to the Global Cancer Statistics 2018 report, colorectal cancer is the fourth leading cause of death, with a mortality rate of 6.1% [1]. In the past, colorectal cancer was considered a high-risk disease in Western countries, but in recent years its incidence is increasing in Asian countries as well. In South Korea, cancer is the most common cause of death, and colorectal cancer is the third most common cause of cancer-related deaths [2]. In addition, the incidence of colon cancer in South Korea has increased significantly over the last 20 years. The incidence of colon cancer was 20.4/100,000 in 1999 and increased to 31.9/100,000 in 2014 [2].

‘Colorectal polyp’ refers to a lesion consisting of neoplastic tissue protruding above the intestinal mucosa. The adenoma corresponding to the highest rate among polyps is known as a precursor of colorectal cancer [3]. Experts suggest that the management of colorectal polyps is necessary to prevent colorectal cancer. Studies involving long-term follow-up of individuals with colorectal polyps revealed that patients with colorectal polyps had a higher risk of developing colorectal cancer than those without polyps. Specifically, the risks of tubular adenomas, tubulovillous adenomas, and villous adenomas increased 1.41-, 2.56-, and 3.82-fold, respectively [4]. Therefore, colorectal polyp prevention is considered to be ultimately associated with colorectal cancer prevention [5].

Risk factors for colorectal polyps and cancer include non-modifiable factors—such as age, sex, and genetics—with a high incidence of polyps being reported in older adults and men [6]. In addition, well-known lifestyle-related health behaviors such as obesity, low physical activity, smoking, and high alcohol intake have been reported to affect the risk of colorectal polyps [7]. In particular, the risk of colorectal polyps increased 2.47-, 1.33-, and 1.40-fold for smoking, drinking, and obesity, respectively [8]. Colonoscopy of 489 Japanese individuals indicated that the prevalence of polyps was 55.0% in men and 38.3% in women, demonstrating an increased risk in men compared to women [9]. Obesity and reduced physical activity are factors that increase the prevalence of polyps, as individuals with body mass index (BMI) > 25.0, exhibit a 1.412-fold increased prevalence compared to those with BMI < 23 [10]. For physical activity, it was reported that the incidence of colorectal polyps decreased by 10% in individuals who exercised for more than 3 h per week and increased by 19% in individuals who were sedentary for more than 70 h per week [11]. A meta-analysis reported a 16% relative risk reduction when high and low activities were compared [7].

Previous research suggests that high physical fitness reduces the incidence of colorectal polyps. In a cardiorespiratory fitness (CRF) analysis in young adult men, the prevalence of colorectal polyps was reduced by 31.3% in individuals in the upper quartile group compared to those in the lower quartile group [12]. However, previous studies included relatively small sample sizes, and studies using cardiorespiratory fitness and leg strength using isokinetic equipment to evaluate patients with colorectal polyps are very rare. Moreover, there are limitations to studies that simultaneously performed analyses—including physical fitness, obesity, and health behavior.

Therefore, this study cross-sectionally analyzed the prevalence of colorectal polyps according to cardiorespiratory fitness, leg muscle strength, obesity, and health behavior among older adults in Asian countries where the incidence of colorectal cancer is increasing. We hypothesized that lower fitness levels, reduced frequency of physical activity, and negative health behavior would be associated with a higher prevalence of colorectal polyps.

## 2. Methods

**Figure 1:**
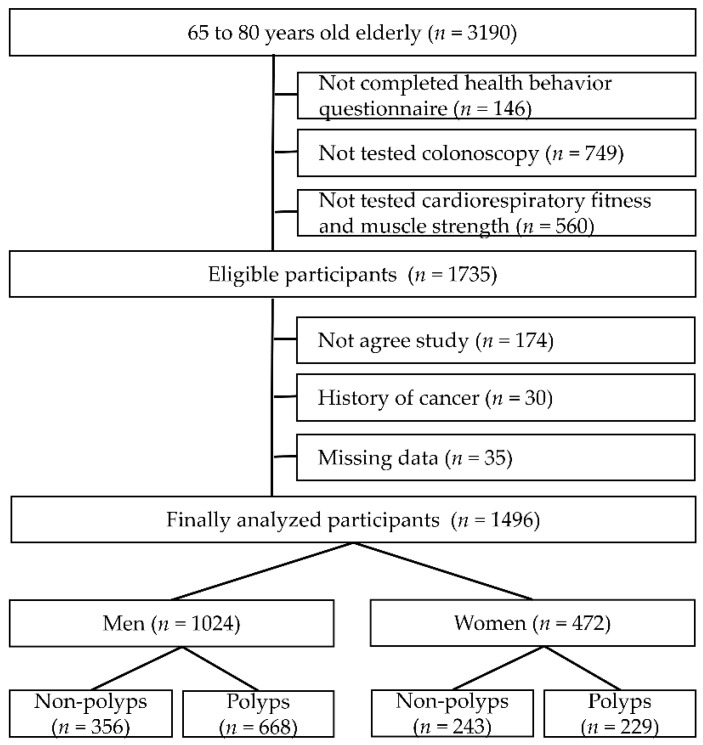
Diagram of participant inclusion and exclusion.

Among the patients who visited the healthcare center at Zhengzhou university affiliated hospital from January 2016 to September 2018, 1496 individuals aged 65–80 years (men: 1024, women: 472) were analyzed. The participants were asymptomatic and visited for disease prevention and care. Healthcare center visitors were generally informed through brochures or bulletin boards, and participate voluntarily. Patients were excluded if they (1) were unable to complete the fitness test due to physical and psychological discomfort or refusal (*n* = 560), (2) had not completed the questionnaire (*n* = 146), (3) had a history of colorectal cancer, and (4) were diagnosed with cancer through biopsy of polyps at colonoscopy (*n* = 30) (Figure 1).

The participants fasted from 6:00 p.m. the day preceding the test. All tests were performed in the morning, and the order of inspection was designed to prevent inter-influence. Center visits were made before 8 a.m. During the visit, a health questionnaire was completed, and physician consultations were conducted. The doctor consulted the medical history and determined the patient’s current condition; if there was no abnormality, medical examinations were performed. The first medical examination included electrocardiography, blood pressure measurements. The body height, body weight, and waist circumference of participants were also measured. Subsequently, CRF, muscle strength, and colonoscopy were performed. After all the tests were completed, the doctor evaluated patients for safety and health issues before being discharged.

Participants signed informed consent, and all data was de-identified in conformance with ethical guidelines. The study was conducted in compliance with the Declaration of Helsinki and was approved by the institutional review board of institutional review board of Zhengzhou University (approved number 20210310, on 3 March 2021).

### 2.1. Health Behavior Questionnaire

The questionnaire was self-completed, and the staff provided assistance only if the participant did not understand a question or requested help. Physical activity, alcohol consumption, and smoking status were measured using questionnaires published by the WHO. Physical activity was investigated using the International Physical Activity Questionnaire (IPAQ), and the weekly frequency of moderate and high intensity was used for analysis [13]. Alcohol consumption was measured using the Alcohol Use Disorders Identification Test (AUDIT) questionnaire, and monthly or weekly frequency was used for analysis [14]. The Global Adult Tobacco Survey (GATS) was used for smoking, and smoking status was analyzed and classified into current, former, and never [15].

### 2.2. Obesity

The WHO Asia–Pacific standards were applied for obesity [16]. BMI ≤ 22.9 kg/m^2^ was classified as normal, BMI of 23.0–24.9 kg/m^2^ as overweight, and BMI ≥ 25.0 kg/m^2^ as obese. A tape measure was used to determine the waist circumference by measuring at the thickest part between the last lip and above the iliac crest. The participant stood with the upper body upright and breathed naturally; the examiner adjusted the tape measure horizontally and recorded the value obtained immediately after the participant exhaled. Care was taken to avoid placing pressure on the skin when using the tape measure. A waist circumference >90 cm in men or >80 cm in women was classified as obesity according to the WHO adult Asian classification [17].

### 2.3. Fitness Test

#### 2.3.1. Cardiorespiratory Fitness

CRF was measured using gas analysis (Vmax 229, SensorMedics Corporation, Yorba Linda, CA, USA). The BRUCE protocol was selected and initiated with light walking; the speed and inclination were gradually increased every 3 min [18]. The examiner provided detailed information regarding the purpose of the examination, advantages, disadvantages, and any discomfort that may occur during the examination. The examination was conducted after obtaining written consent from the participants. Participants who had no experience using a treadmill performed the test after sufficient practice and recovery. One minute before each stage change, the heart rate, blood pressure, electrocardiogram status, subjective perception of difficulty, and chest pain were recorded. The examination was continued until the participant requested to stop; if there were a clinically significant abnormality in the heart, blood pressure, and heart rate, the test was discontinued by the examiner, and those data were excluded from the analysis.

O_2_ and CO_2_ ventilated through the sensor were measured in real time, and the test was continued until the participant stopped. The maximum oxygen uptake obtained during this process was recorded in mL/kg/min. During the test, a 12-lead electrocardiogram (Case8000, GE Marquette Co., Milwaukee, WI, USA) was used to evaluate heart function, and all tests were conducted under the supervision of a cardiologist.

#### 2.3.2. Leg Muscle Strength

An isokinetic dynamometer (CSMi) and HUMAC software (CSMi HUMAC NORM, Stoughton, MA, USA) were used to measure leg muscle strength. Before the test, participants were asked to confirm their physical condition, whether they had injuries other than the knee, and if they could exert maximum strength. The participants performed 15 min of warm-up exercises including cycling, walking, and stretching. The examiner explained what the test entailed and conducted appropriate practice sessions. Before initiating the actual test, participants performed several movements at low, medium, and high speeds to adapt to and gain familiarity with the test equipment. After sufficient practice, the actual test was performed.

Concentric knee extension and flexion were measured at 60°/s. The test range was 0° (knee extension) to 90° (flexion), and the starting posture was 90°. The participant fixed his or her body with a strap while sitting on a chair and used both hands to grip the handle. When the examiner provided the start signal, extension was performed first, followed by flexion. The peak torque (N·m) of extension and flexion were recorded, and the values were summed [19]. Muscle strength is affected by body weight; therefore, the value used in the analysis was calculated by dividing the peak torque obtained by the body weight (N·m/kg·m^−1^).

### 2.4. Colonoscopy

For colonoscopy, the participants fasted from 6:00 p.m. the day preceding the examination, and antiplatelet and anticoagulant drugs were discontinued 3 days before the examination. Polyethylene glycol electrolyte (Coolprep Powder, Taejoon Pharm, Seoul, Korea) and water were orally administered to clean the colorectal. The ingredients are as follows: (A) Each pack (56.402 g) consisted of polyethylene glycol 3350 (50 g), potassium chloride (0.5075 g), sodium chloride (1.3455 g), and anhydrous sodium sulfate (3.75 g). (B) Each pack (5.3 g) contained ascorbic acid (2.35 g) and sodium ascorbate (2.95 g). For the first dose, from 8:00 p.m. to 9:00 p.m. the day before the test, packs A and B were taken twice with 500 mL of water. The second dose was taken twice from 4:00 a.m. to 5:00 a.m. on the day of the medical test. For endoscopy, conscious sedation colonoscopy was performed by a gastroenterologist. For participants with colorectal polyps, the type, location, and size were measured, polyps were removed using biopsy forceps or polypectomy, and tissue analysis was performed to identify malignant or benign polyps.

### 2.5. Data Analysis

Analyses were performed using SPSS (version 25.0; IBM SPSS Inc., Armonk, NY, USA). CRF was recorded and analyzed in mL/kg/min, and muscle strength (Nm) was recorded. Muscle strength is affected by body weight (BW); therefore, relative values (kg/BW and Nm/BW) were used in the analyses. The groups were divided into tertiles and classified as high, middle, or low.

General characteristics were expressed as medians and standard error, and normality tests were performed for both sexes using the Kolmogorov–Smirnov test. For both men and women, the variables did not follow a normal distribution and the Mann–Whitney U test was performed for between-group comparisons. Furthermore, polyp and non-polyp groups were compared using a nonparametric method for non-normal distribution.

Relationships between categorical variables, physical activity, alcohol consumption, smoking, and colorectal polyps were examined using the chi-square test, and the prevalence of colorectal polyps was analyzed using logistic regression to calculate the odds ratios (ORs). Multiple regression and stepwise analyses were conducted to select adjustment variables, including CRF, muscle strength, smoking status, and waist circumference. Statistical significance was set at *p* < 0.05.

## 3. Results

Table 1 summarizes the general characteristics of the participants. There were significant differences between men and women regarding height (*p* < 0.001), weight (*p* < 0.001), BMI (*p* = 0.009), waist circumference (*p* < 0.001), CRF (*p* < 0.001), and muscle strength (*p* < 0.001). There were also significant differences between men and women in terms of smoking, alcohol consumption, and physical activity (*p* < 0.05).

Table 2 summarizes the results of the chi-square test. The proportion of colorectal polyps was analyzed according to independent variables. In men, there were significant differences in waist circumference (*p* = 0.006), CRF (*p* < 0.001), muscle strength (*p* = 0.004), smoking (*p* < 0.001), alcohol consumption (*p* = 0.046), and physical activity (*p* = 0.021) among individuals with and without polyps. In women, there were also significant differences in waist circumference (*p* < 0.001), CRF (*p* < 0.001), muscle strength (*p* = 0.038), and physical activity (*p* = 0.044) in the polyp and non-polyp groups.

**Figure 2:**
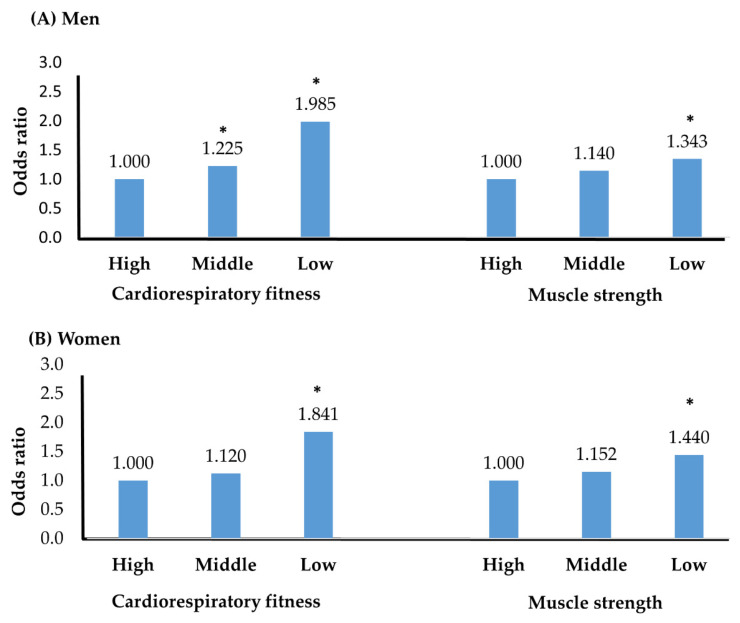
Prevalence of colorectal polyps according to fitness level. (**A**) men’s odds ratio; (**B**) women’s odds ratio; * p < 0.05; adjusted variables included age, cardiorespiratory fitness, muscle strength, smoking, income, physical activity for men, and age, cardiorespiratory fitness, muscle strength, and waist circumference for women.

To analyze the prevalence of colorectal polyps based on physical fitness, patients were divided into low, middle, and high physical fitness groups according to tertiles. In men, the ORs for low CRF and low muscle strength were 1.985 and 1.343 (*p* < 0.05), whereas in women, they were 1.841 and 1.440, respectively (*p* < 0.05) (Figure 2).

A survey was conducted to assess participants’ health behaviors. Compared to normal weight, the OR for obesity was 1.151 (95% CI, 1.010–2.291, *p* = 0.014) in men. The OR for smoking was 1.884 (95% CI, 1.298–2.749, *p* < 0.001). The OR for physical activity was 1.693 (95% CI, 1.087–2.913, *p* = 0.022) after age adjustment; however, no significant value was derived in the multivariate adjustment (Table 3).

Table 4 shows the prevalence of colorectal polyps among women. Compared to normal weight, the OR for obesity was 1.178 (95% CI, 1.015–2.612, *p* = 0.019). Smoking did not significantly increase the OR compared to non-smokers. Compared to daily physical activity, the OR for lack of physical activity was 1.861 (95% CI, 1.068–3.451, *p* = 0.018).

## 4. Discussion

Globally, cancer has a high mortality rate, and colorectal cancer has the third-highest incidence among the different types of cancers [20]. Although colorectal polyps are not always cancerous, they are reported precursors in colorectal carcinogenesis, and the incidence of colorectal cancer is high in individuals with colorectal polyps [4]. The biological mechanisms underlying colorectal polyps have not been fully elucidated.

This study analyzed the prevalence of colorectal polyps in the elderly based on CRF, muscle strength, and abdominal obesity, as well as health behaviors—particularly smoking status, alcohol consumption, and physical activity.

The results of this study indicated that colorectal polyps were more prevalent in men and women with lower CRF. A previous study similarly reported that a high CRF reduced the incidence of colorectal polyps in men by 31.3% [12]. Nakamura et al. [21] reported that colorectal polyps exhibited an inverse relationship with CRF. Studies on CRF and polyps are still few compared to cancer studies, and some studies showed that the presence of association differs according to age and sex. A large cohort study in which the incidence of various cancers according to CRF was assessed over 20 years revealed that CRF was significantly associated with proximal colorectal cancer and lung and bladder cancer. Furthermore, the risk of proximal colorectal cancer in the middle CRF group was 70% lower than that in the low CRF group [22]. Takemura et al. [23] reported that the group with high endurance fitness had a lower the prevalence of colon polyps by 64.0% compared to the group in the low fitness in middle-aged men. Meanwhile, the same study did not show any significant results according to fitness in middle-aged and elderly individuals. Moreover, a UK study revealed that the risk of colorectal cancer was lowered by 28% in men with high CRF, but not significantly in women [24].

Although the exact biological mechanisms supporting the association between CRF and colorectal cancer or polyps have not been clearly established, several hypotheses exist. Cardiorespiratory function is inversely proportional to fasting insulin levels and positively related to insulin sensitivity. Thus, high CRF blocks the pathway that contributes to the formation of neoplastic tumors [25]. Next, training to increase CRF reduces interleukin-6 and TNF-α levels and increases interleukin-10 levels, creating an anti-inflammatory environment [26]. In addition, the higher the fitness level, the higher the antioxidant enzymes, which increases recovery from oxidative stress [27].

The results of muscle strength shown in this study were also approached in a similar context to CRF. Recently, research on sarcopenia has been actively conducted, and this condition is regarded as a general phenomenon associated with aging. Sarcopenia is diagnosed based on muscle mass and grip strength. A study conducted in Korea reported a significant relationship between colorectal neoplasia and sarcopenia. There was a significant difference in the incidence of sarcopenia (51.1%) compared to that in the healthy group (41.1%) [28]. In a study that evaluated grip strength, older patients with ≥5 polyps exhibited significantly lower grip strength than patients with <5 polyps [29].

In this study, the results showed that the prevalence of colorectal polyps was lower in men and women with high physical activity. This fact was also mentioned in the representative health benefits of physical activity published by the American College of Sports Medicine [18]. Moreover, a previous study demonstrated that high physical activity results in a low risk of colorectal polyps. Wolin et al. [30] reported an inverse relationship between physical activity and colorectal polyps and reported that the OR for polyps in the physical active group was 16% lower than that in the inactive group. Additionally, another study reported that ~15% of colorectal cancers can be prevented through physical activity; however, the type, intensity, and frequency of physical activity that are required to prevent colorectal polyps is still unclear [31]. In a similar study, the high-frequency physical activity group showed a 25.7% lower incidence of polyps than the low-frequency physical activity group [12].

The probable mechanisms of colorectal cancer prevention through physical activity are related to insulin levels, inflammation, myokine levels, and immune responses. In particular, physical activity can potentially reduce toxins generated during food digestion by decreasing the time that food remains in the large intestine; however, these mechanisms are still being debated [31]. Some studies have shown that physical activity does not affect polyp occurrence. In one study, intensive physical activity did not exhibit a significant relationship with the occurrence of polyps [32]. Conversely, another study revealed a significant relationship between the frequency and intensity of physical activity in young adults and middle-aged patients and the presence of polyps, whereas there was no significant relationship in older individuals [12]. Therefore, long-term follow-up studies with larger cohorts are required.

Insulin has been implicated in colorectal tumor cell development; therefore, physical activity may reduce the risk of cancer by reducing insulin resistance and insulin levels, which impacts the insulin-like growth factor pathway [33]. Inflammation contributes to the onset and progression of cancers and polyps. Exercise reduces systemic inflammation and improves immune function. Physical activity has been suggested to prevent the occurrence of polyps by reducing interleukin-6 and C-reactive protein [34,35]. Myokine secretion by muscles increases insulin sensitivity through cytokines and decreases pro-inflammatory cytokine production. Typically, secreted protein acidic and rich in cysteine is released from muscle tissue after exercise and increases apoptosis, which may inhibit colorectal neoplastic development [36]. Finally, the immune system affects the neoplastic cells in the body. It has been reported that the accumulation of macrophages in tumors causes colorectal tumor development, and that exercise reduces the expression of macrophages and regulatory T-cell markers [37,38]. Additionally, cell proliferation and apoptosis, antioxidant capacity, and DNA repair mechanisms exist; however, they are still being studied, and the exact mechanisms remain controversial [39].

In this study, the prevalence of colorectal polyps in men was higher than that in women. Similar results have been reported in other studies. In a study conducted in Japan, the incidence of adenomatous polyps was 55.0% in men and 38.3% in women [9], and a study of older adults in the United States reported a 10.2% higher incidence in men than in women [40]. However, a Chinese study reported a similar prevalence of adenoma polyps between the sexes (men: 53.7%, women: 49.2%) [41]. Various studies are still ongoing on the reasons for the high incidence of colorectal cancer and polyps in men, but these results remain inconclusive. Pathogenesis involves sex-specific physiologic studies of epigenetics, tumor suppressor TP53 gene, and sex-specific hormones—such as estrogen, estradiol, testosterone, and sex hormone binding globulin [42,43].

More clearly, men have higher tobacco use and alcohol intake than women [44]; these results are also shown in this study. Previous studies have reported that drinking and smoking are associated with a higher incidence of polyps or cancer [5,7]. In the present study, a high prevalence of polyps was reported in men who were current smokers. Although the association between smoking and the occurrence of colorectal polyps is sometimes not significant [45], smoking has long been considered a risk factor for colorectal polyps. In a study by Fliss–Isakov et al. [46], the prevalence of polyps in current smokers was 3.15 times that in the group that never smoked; the prevalence in the former smoking group was 1.51 higher than that in the group that never smoked, even 1 year after smoking cessation. The OR for those who smoked for >20 years was 3.26 times that for non-smokers, and for those who smoked ≥10 cigarettes per day, the OR exhibited a 2.13-fold increase. In a study by Ijspeert et al. [32], the OR of smokers was 4.5 times that of former smokers or persons that never smoked. It is not clear how the negative effects of smoking affect colorectal polyps or colorectal cancer, but it is most likely that smoking promotes cancer through the association of environmental carcinogens such as ultraviolet radiation, free oxygen species, and various chemicals [46].

Drinking alcohol is considered a major activity affecting health, along with smoking and physical activity; however, this study did not identify any significant relationship between alcohol consumption and polyp occurrence in women, similar to the results of other studies. Shrubsole et al. [47] reported that alcohol consumption was not strongly associated with the incidence of colorectal polyps, similar to smoking. Ijspeert et al. [32] reported a significant relationship with smoking, but no significant association with alcohol consumption. In a study in China, smoking increased the risk 1.35 times, whereas alcohol consumption did not exhibit a significant effect [48]. Nevertheless, in a Korean study, the OR for advanced adenoma in individuals who consumed alcohol was 2.697 times that of individuals who did not consume alcohol [49]. In a study applying the WHO guidelines, it was reported that the prevalence of polyps among those who consumed alcohol increased 1.772-fold in high-risk middle-aged men compared to that in low-risk individuals; however, there were no significant differences in older individuals in the same study [44]. Therefore, although the studies did not achieve consistent results, high alcohol consumption was clearly considered to have negative effects on colorectal health. The inconsistent results may due to the complexity in the type of measurement, amount, and frequency of drinking in surveys [50].

It has been reported that alcohol influences neoplasia through various pathways. Typically, metabolites such as acetaldehyde are produced, and these metabolites can promote carcinogenesis. These mechanisms induce genetic, acquired, biochemical, and immunological abnormalities, leading to chronic inflammation and the formation of neoplastic lesions [50]. There are multiple pathways and factors that induce neoplastic substances, and there is ongoing research on the underlying mechanisms; however, more research is still required.

Abdominal obesity has traditionally been widely applied in cardiovascular disease research and is considered a major risk factor. In this study, the relationship with colorectal polyps was investigated, and the prevalence of polyps was increased in both men and women with obesity. These results are similar to those of various studies. Kim et al. [51] reported that abdominal obesity increased the prevalence of colonic adenoma by 2.77 times for men and 2.65 times for women compared to those with normal waist circumference. In the study of men’s waist circumference divided by quartiles, the prevalence in the second quartile increased by 1.20 times, 1.45 times in the third quartile, and 1.64 times in the fourth quartile compared to the first quartile [52]. Several mechanisms have been proposed to explain the pathway by which abdominal obesity increases the risk of colorectal neoplasia [53,54]. It is commonly argued that a variety of inflammatory cytokines are released from adipose tissue, including the cytokines tumor necrosis factor and IL-6 [53]. In addition, the accumulation of visceral fat is a strong determinant of insulin resistance and hyperinsulinemia [54].

This study emphasizes the need for physical activity, physical fitness, and active health care for the prevention of colorectal polyps and extension of colorectal cancer. Therefore, the greatest strength of this study is that the prevalence of colorectal polyps was evaluated by examining muscle strength, physical activity, and health habits. Despite these strengths, this study has several limitations. Since this was a cross-sectional study, there was a limit to determining a causal relationship. Diverse types of polyps—such as adenoma, hyperplastic, serrated, and hamartomatous polyps—have been analyzed in another study [55]. However, we did not assess the type, shape, or number of polyps. Although cancer is known to be highly affected by genetics, our study did not investigate family history, and since it was conducted at a single center, regional characteristics could not be confirmed.

Several studies have examined the incidence of CRC based on the socioeconomic status of the participants. Those with low education and low income exhibited 42% and 31% increased risk of colorectal cancer compared to those with higher education and higher income, respectively [56]. This indicates that individuals with higher income or education spend more time and money on health care, live in areas with better access to medical care, and obtain tests early when they have health problems [57]. In this study, socioeconomic status was investigated, but since the focus was on physical fitness and health behavior, analyses pertaining to social factors were not performed.

In addition to the risk factors investigated in this study, various other factors influence the prevalence of colorectal polyps. For example, diabetes was associated with colorectal polyps [58], and shift workers have been reported to exhibit a higher prevalence of polyps than non-shift workers [45]. The scope of the health behavior survey was limited to physical activity, alcohol consumption, and smoking, and nutrition surveys were excluded from this study.

For the prevention of colorectal health, lifestyle modification in terms of health behaviors is important, and experts recommend regular colonoscopy, even for asymptomatic individuals [59]. Therefore, further research to elucidate the underlying mechanism of polyp formation according to exercise intensity and physical fitness level will be more meaningful if a larger number of participants are recruited, analyzed based on the various polyp types, and followed longitudinally over the long term.

## 5. Conclusions

In this cross-sectional study, the incidence of colorectal polyps in the elderly was higher in men than in women. Low cardiorespiratory fitness increased the prevalence of colorectal polyps 1.985- and 1.841-fold in men and women, respectively. Moreover, abdominal obesity, low physical activity, and low muscle strength also increased the prevalence of colorectal polyps in both men and women. Older men with current tobacco use and high alcohol consumption frequency increased the risk of polyp formation. Therefore, high CRF, high physical activity, and normal abdominal circumference may have a role in preventing colorectal polyps in the elderly.

## Figures and Tables

**Table 1:** General characteristics of participants.

Variables	Men (*n* = 1024)	Women (*n* = 472)	*p*-Value
Age, years	68.0 (0.295)	68.3 (0.414)	0.522
Height, cm	168.0 (0.568)	153.0 (0.540)	<0.001 *
Weight, kg	70.4 (0.839)	58.7 (0.839)	<0.001 *
BMI, kg/m^2^	25.3 (0.849)	24.5 (0.319)	0.009 *
Waist circumference, cm	86.0 (0.705)	79.0 (0.985)	<0.001 *
Cardiorespiratory fitness, mL/kg ^−1^/min^−1^	29.7 (0.492)	25.3 (0.518)	<0.001 *
Muscle strength, N·m/kg·m^−1^	2.90 (0.08)	1.92 (0.07)	<0.001 *
Smoking status, *n* (%)			
Never	182 (18.3%)	498 (88.3%)	<0.001 *
Former	462 (46.4%)	34 (6.0%)	
Current	352 (35.3%)	32 (5.7%)	
Alcohol frequency, *n* (%)			
0–1 day week	426 (42.8%)	498 (88.3%)	<0.001 *
2–3 days/week	274 (27.5%)	52 (9.2%)	
4–7 days/week	296 (29.7%)	14 (2.5%)	
Physical activity, *n* (%)			
Every day	143 (14.4%)	80 (14.2%)	<0.001 *
3–5 days/week	316 (31.7%)	170 (30.1%)	
1–2 days/week	337 (33.8%)	150 (26.6%)	
None	200 (20.1%)	164 (29.1%)	
Education, *n* (%)			
Graduate	180 (18.1%)	33 (5.9%)	<0.001 *
College or university	379 (38.1%)	150 (26.6%)	
High school	437 (43.9%)	381 (67.6%)	
Income (USD), *n* (%)			
>9000 USD	187 (18.8%)	95 (16.8%)	<0.001 *
3000 to 9000 USD	536 (53.8%)	207 (36.7%)	
<3000 USD	273 (27.4%)	262 (46.5%)	
Colorectal polyp, *n* (%)	668 (65.2%)	229 (48.5%)	<0.001 *

* p < 0.05; Data were expressed as median (standard error) or number (%). Abbreviations: BMI, body mass index; BW, body weight.

**Table 2:** Incidence of colorectal polyps associated with different variables.

Variables	Men		*p*-Value	Women		*p*-Value
	Non–Polyp	Polyp		Non–Polyp	Polyp	
Waist circumference						
Normal	257 (74.1%)	425 (65.5%)	0.006 *	207 (71.4%)	173 (63.1%)	<0.001 *
Obesity	90 (25.9%)	224 (34.5%)		83 (28.6%)	101 (36.9%)	
Cardiorespiratory fitness						
High	153 (44.1%)	188 (29.0%)	<0.001 *	121 (41.7%)	73 (26.6%)	<0.001 *
Middle	118 (34.0%)	212 (32.7%)		106 (36.6%)	80 (29.2%)	
Low	76 (21.9%)	249 (38.3%)		63 (21.7%)	121 (44.2%)	
Muscle strength						
High	135 (38.9%)	195 (30.0%)	0.004 *	113 (39.0%)	79 (28.8%)	0.038 *
Middle	116 (33.4%)	216 (33.3%)		90 (31.0%)	96 (35.1%)	
Low	96 (27.7%)	238 (36.7%)		87 (30.0%)	99 (36.1%)	
Smoking status						
None	75 (21.6%)	107 (16.5%)	<0.001 *	268 (92.5%)	230 (84.0%)	0.107
Former	179 (51.6%)	283 (43.6%)		12 (4.1%)	22 (8.0%)	
Current	93 (26.8%)	259 (39.9%)		10 (3.4%)	22 (8.0%)	
Alcohol frequency						
0–1 day/week	156 (45.0%)	270 (41.6%)	0.046 *	256 (88.3%)	242 (88.4%)	0.154
2–3 days/week	109 (31.4%)	165 (25.4%)		30 (10.3%)	22 (8.0%)	
4–7 days/week	82 (23.6%)	214 (33.0%)		4 (1.4%)	10 (3.6%)	
Physical activity						
6–7 days/week	56 (16.1%)	87 (13.5%)	0.021 *	43 (14.8%)	37 (13.5%)	0.044 *
3–5 days/week	121 (34.9%)	195 (30.0%)		95 (32.8%)	75 (27.4%)	
1–2 days/week	118 (34.0%)	219 (33.7%)		73 (25.2%)	77 (28.1%)	
None	52 (15.0%)	148 (22.8%)		79 (27.2%)	85 (31.0%)	

* p < 0.05; data are expressed as number (%).

**Table 3:** Prevalence of colorectal polyp in men based on health behavior.

Variables	Model 1		Model 2	
	OR (95% CI)	*p*-Value	OR (95% CI)	*p*-Value
Waist circumference				
Normal	Reference	–	Reference	–
Obesity	1.254 (1.017–1.861)	0.023 *	1.151 (1.010–2.291)	0.014 *
Smoking status				
None	Reference	–	Reference	–
Former	1.100 (0.847–1.598)	0.410	1.098 (0.857–1.597)	0.460
Current	1.781 (1.349–2.856)	<0.001 *	1.884 (1.298–2.749)	<0.001 *
Alcohol frequency				
0–1 day/week	Reference	–	Reference	–
2–3 days/week	1.044 (0.664–1.267)	0.413	1.019 (0.660–1.161)	0.271
4–7 days/week	1.541 (0.545–2.219)	0.084	1.121 (1.061–2.113)	0.034 *
Physical activity				
Every day	Reference	–	Reference	–
3–5 days/week	1.074 (0.686–1.721)	0.687	1.039 (0.709–1.569)	0.581
1–2 days/week	1.114 (0.741–1.796)	0.749	1.241 (0.798–1.880)	0.145
None	1.397 (1.018–2.340)	0.019 *	1.693 (1.087–2.913)	0.022 *

* p < 0.05; Abbreviations: OR, odds ratio; CI, confidence interval; Adjusted variables: Model 1, age; Model 2, age, cardiorespiratory fitness, muscle strength, smoking, and income.

**Table 4:** Prevalence of colorectal polyp in women based on health behavior.

Variables	Model 1		Model 2	
	OR (95% CI)	*p*-Value	OR (95% CI)	*p*-Value
Waist circumference				
Normal	Reference	–	Reference	–
Obesity	1.231 (1.014–2.019)	0.021 *	1.178 (1.015–2.612)	0.019 *
Smoking status				
None	Reference	–	Reference	–
Former	1.358 (0.584–2.513)	0.126	1.040 (0.653–3.746)	0.294
Present	2.769 (0.735–5.570)	0.108	2.591 (0.747–4.710)	0.200
Alcohol frequency				
0–1 day/week	Reference	–	Reference	–
2–3 days/week	0.889 (0.534–1.840)	0.667	0.879 (0.348–1.640)	0.613
4–7 days/week	3.145 (0.646–7.687)	0.124	2.491 (0.640–5.749)	0.314
Physical activity				
Every day	Reference	–	Reference	–
3–5 days/week	0.851 (0.547–1.904)	0.850	1.671 (0.870–2.501)	0.349
1–2 days/week	1.119 (0.639–1.841)	0.521	1.519 (0.744–3.697)	0.870
None	1.150 (0.841–2.409)	0.221	1.861 (1.068–3.451)	0.018 *

* p < 0.05; OR, odds ratio; CI, confidence interval; adjusted variables: Model 1, age; Model 2, Model 1, age; Model 2, age, cardiorespiratory fitness, muscle strength, and waist circumference.

## Data Availability

The data are not publicly available because of privacy or ethics.
